# Identifying work ability promoting factors for home care aides and assistant nurses

**DOI:** 10.1186/1471-2474-13-1

**Published:** 2012-01-11

**Authors:** Agneta Larsson, Lena Karlqvist, Mats Westerberg, Gunvor Gard

**Affiliations:** 1 Department of Health Sciences, Luleå University of Technology, SE-97187 Luleå, Sweden; 2 Department of Business Administration, Technology and Social Sciences, Luleå University of Technology, SE-97187 Luleå, Sweden; 3 Department of Health Sciences, Lund University Hospital, SE-221 85 Lund, Sweden

## Abstract

**Background:**

In workplace health promotion, all potential resources needs to be taken into consideration, not only factors relating to the absence of injury and the physical health of the workers, but also psychological aspects. A dynamic balance between the resources of the individual employees and the demands of work is an important prerequisite. In the home care services, there is a noticeable trend towards increased psychosocial strain on employees at work. There are a high frequency of work-related musculoskeletal disorders and injuries, and a low prevalence of sustainable work ability. The aim of this research was to identify factors promoting work ability and self-efficacy in care aides and assistant nurses within home care services.

**Methods:**

This study is based on cross-sectional data collected in a municipality in northern Sweden. Care aides (n = 58) and assistant nurses (n = 79) replied to a self-administered questionnaire (response rate 46%). Hierarchical multiple regression analyses were performed to assess the influence of several independent variables on self-efficacy (model 1) and work ability (model 2) for care aides and assistant nurses separately.

**Results:**

Perceptions of personal safety, self-efficacy and musculoskeletal wellbeing contributed to work ability for assistant nurses (R^2^adj of 0.36, *p *< 0.001), while for care aides, the safety climate, seniority and age contributed to work ability (R^2^adj of 0.29, *p *= 0.001). Self-efficacy was associated with the safety climate and the physical demands of the job in both professions (R^2^adj of 0.24, *p *= 0.003 for care aides), and also by sex and age for the assistant nurses (R^2^adj of 0.31, *p *< 0.001).

**Conclusions:**

The intermediate factors contributed differently to work ability in the two professions. Self-efficacy, personal safety and musculoskeletal wellbeing were important for the assistant nurses, while the work ability of the care aides was associated with the safety climate, but also with the non-changeable factors age and seniority. All these factors are important to acknowledge in practice and in further research. Proactive workplace interventions need to focus on potentially modifiable factors such as self-efficacy, safety climate, physical job demands and musculoskeletal wellbeing.

## Background

In the workplace, health can be seen as a dynamic balance between personal resources and factors related to the workplace [1]. Similarly the concept of 'work ability' can be reflective of a balance between a person's resources and the demands of their work [1-3], where the former is linked to health and functional abilities, values, attitudes, education, work skills and health practices [2,4], and the latter to the actual content, demands and organisation of work, as well as the working environment [2,3]. In workplace health and safety promotion, all of the potential resources related to work should be taken into consideration, not only physical health and factors associated with the absence of injury; the aspects taken into account should include psychological ones [4,5]. The organisational, psychological, social and physical requirements for health and safety need to be given increased attention and priority in proactive interventions [1,6].

It has been shown that many workers underestimate their actual risk of developing work-related musculoskeletal disorders (WMSDs) [7]. Perceiving oneself to have a vulnerability to a work-related injury or illness, this can help to motivate a behavioural change and the adoption of safer work behaviour. That is, when combined with a perceived ability to take control over the risk factors at work [7,8]. The process through which individuals gain greater control over the actions affecting their health is related to 'self-efficacy', i.e. a belief in one's own ability to overcome obstacles and adopt the behaviour one desires. Perceived self-efficacy in performing physical tasks, fulfilling role expectations, prioritising health and safety at work and managing musculoskeletal disorders is important for a person's health, safety and work ability [9-11]. Self-efficacy is a key concept in Social cognitive theory, emphasising the reciprocal interaction between personal resources, behavioural capability, and external environmental factors [12]. Thus, self-efficacy is also influenced by external factors that are primarily under the control of others in the work organisation. For example situational constraints, the social support available, and the actual opportunities available to workers to exert control over decisions and actions [13,14]. Job control and social support are well-known determinants of good health, as clarified by Karasek and Theorell in the development of the demand-control-support model [15-17]. Recently, this model was applied in research on workplace safety [18,19]. The quality of the social support and the direction it takes can be further specified. Such as the perceptions of a good 'safety climate', that can encourage employees in the performance of their work safely [18,20]. Safety climate can be described as the shared perceptions of members in a social unit of safety-related policies and practices; for example, communication between peers relating to safety, commitment to and the priority of safety issues, the refusal to accept risk, and the ability of management to manage and prioritise safety [21]. In addition to having the potential to support specific kinds of behaviour and to ensure certain safety-related outcomes, measured, for example, in terms of low injury rates [20], the social environment can play a role in supporting the work ability of its members [2]. The safety climate and its relation to safe behaviour has recently begun to be explored in medical care sectors [22,23].

**Figure 1:**
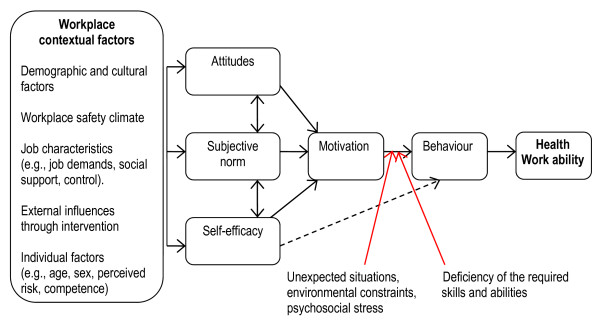
**A model showing factors important for an adequate safety behaviour and good health and work ability, adapted from Fishbein **[24]**and modified by Larsson, Karlqvist and Gard **[25].

An integrative model linking attitudes, behavioural control and behaviours was recently proposed by Fishbein [24]. We found this model useful and modified it by adding health and work ability as the outcome measures and by specifying important work place contextual factors according to the literature (Figure 1) [25]. The model proposes that factors in the society and at the work place can influence the individual employee's attitudes, perceived norms and self-efficacy beliefs. Also the influences of individual background factors are shown. All these factors can influence the level of motivation in relation to a desired outcome, for example a safe work behaviour, musculoskeletal wellbeing and work ability. Also factors that may hinder the person to perform his/her intended action are addressed, such as unexpected events and lack of specific skills.

The combination of an aging population and financial limitations in the medical care sector are placing high demands on the municipal home care services. In association with this, a trend towards increased psychosocial strain at work can be noted amongst front-line home care staff [26], with a high frequency of WMSDs and injuries, and a low prevalence of having a sustainable work ability [26-28]. In the medical care sector, a disparity between the working conditions has been noted for different professions in the same medical units [29,30], suggesting that similar profession-related differences might be present among municipal home care front-line workers. Power structures and group compositions according to age, gender, ethnicity, class and educational level, determine the distribution of power among work unit members [29,31]. Within medical care, employees with a lower degree of autonomy, self-efficacy and authority to influence the job content had the highest overall physical exposure levels [30]. Health and work ability promoting factors among employees in human relation professions within the public sector in Sweden have recently begun to be studied. Clear work tasks, positive feedback, physically non-strenuous work, self-rated health and leisure time factors were found be important for high work ability [32,33]. Decision latitude, opportunities for learning and development and trust were associated with good health [34]. For home care staff, both the level of self-rated medical and ergonomic knowledge and work related exhaustion were associated with work satisfaction [35]. Unfortunately, care aides and assistant nurses are usually considered together, as one group, in the literature. Both groups assist the service recipient with the performance of social activities, general care and transfers, general house cleaning, and perform delegated medical tasks. However, the groups differ in educational level. Three year of education (in the form of theoretical and practical training in social and medical care) in upper secondary school is required to become an assistant nurse, whereas the training for a care aide is given on the job [35]. Differences in educational level and status may influence how they actively perform their daily work, and also imply differences in working conditions. The aim of this research was to identify factors promoting work ability and self-efficacy in care aides and assistant nurses within home care services in a municipality in northern Sweden.

## Methods

### Population, procedure, and ethics

The research presented here was based on cross-sectional data collected in early 2009 in a northern Swedish municipality as part of a larger health and safety promotion project. In summary, the aim of the total project was to promote health and work ability among the home care staff, by identifying areas for improvement of proactive risk management in home care services, and to implement prioritised changes. In this municipality, a total of 350 care aides and assistant nurses provide home care services to about 900 elderly people (clients) living in private homes. In terms of organisation, the staff members are divided into 18 units, which are managed by 16 supervisors and one head of home care services.

Of the total population of 350, 298 home care workers met the inclusion criterion of having worked in the same unit for the last 6 months and were, therefore, invited to participate in the study. These potential participants were provided with a letter containing information, a letter of consent for them to sign, a hard-copy of the questionnaire and a prepaid envelope, by means of their supervisors. After one reminder, 158 (53%) had returned their questionnaire, however, only 137 (46%) respondents had completed all of the questions required for this study and, thus, were included in the data analysis. The study was performed in compliance with the ethical principles of the Helsinki Declaration, and was approved by the Committee of Research Ethics at Umeå University, Sweden (Dnr 08-217 Ö).

### Definitions of prognostic factors and work ability

Data were obtained through the completion of a comprehensive self-administered questionnaire. The scales used to measure the variables are listed below.

#### Basic background factors

These were obtained from items relating to age (years), sex, profession and seniority (years in post) derived from the QPS Nordic-ADW [36] and had been adjusted by us to the home care services setting.

#### Job-related factors

*Safety climate *was measured using the 50 items of the Nordic Safety Climate Questionnaire (NOSACQ-50), graded on four-point scales with the end points 1 = 'fully disagree' and 4 = 'fully agree'. The questionnaire presumes the respondent to be able to provide a representative voice for a social unit's shared perceptions of the safety climate at the management and work-unit levels [21]. We used a mean value of the seven original dimensions of the safety climate to estimate the respondent's overall impression of the safety climate.

*Psychosocial job demands *were measured using five items derived from the Swedish version of the Job Content Questionnaire [15]. These assessed the requirement to work fast, hard, and using a considerable amount of effort, as well as the impact of not having enough time to do the job and of having to face conflicting demands at work. Again, these were graded on four-point scales with the end points 1 = 'never' and 4 = 'often'. *Physical job demands*, meaning the perceived physical exertion required when performing the job as a whole an ordinary working day, were measured using the Borg RPE scale ranging from 6 = 'very, very low' to 20 = 'very, very high' [37].

#### Individual resources

*Degree of personal safety *was measured using three items graded on a five-point scale: general level of safety at work (end points 1 = 'very bad' and 5 = 'excellent') derived from Olsen [23], the probability of suffering a work-related illness or injury (end points 5 = 'low probability' and 1 = 'high probability'), and if the respondent feels worried and unsafe when thinking about risks at work (end points 5 = 'not worried and unsafe' and 1 = 'very worried and unsafe'), modified from Rundmo [38]. *Personal safety behaviour *was measured using six items on a seven-point scale with the end points 1 = 'never' and 7 = 'always', reflecting the respondent's compliance with personal protection regulations [39]. *Self-efficacy in relation to work and safety *was measured using five items graded on a five-point scale (end points 1 = 'fully disagree' and 5 = 'fully agree') reflecting the respondent's capacity to handle most situations at work, to manage the work tasks as well as peers, having a positive attitude at work, and adjusting work tasks to match his/her capacity, as derived from the QPS Nordic-ADW [36], and one question on being able 'to influence safety' at work.

#### Musculoskeletal wellbeing

*Musculoskeletal wellbeing *during the previous month was measured using seven items on a five-point scale ranging from 1 = 'every day' to 5 = 'very seldom or never' experiencing pain. Each item represented one area of the body; in upper part of back or neck; in lower back; in shoulders or arms; in hands or wrists; in hips; in knees; in feet or ankles [40]. The ratings for each item was summarised and divided by seven to produce an index variable ranging from one to five.

#### Work ability

*Work ability *was measured with three items derived from the Work Ability Index on the present work ability in relation to the physical and mental demands of the job (five-point scale ranging from 1 = 'very bad' to 5 = 'very good'), and on the respondent's own prediction about his or her ability to perform the work that he/she are performing at that time in a further 2 years' time when his/her health is taken into consideration (response alternatives 1 = 'no, unlikely, 4 = 'not certain' or 7 = 'yes, most likely) [41]. The ratings for each item were summarised to produce an index variable ranging from 3 to 17.

### Data analysis

Analyses were performed separately for the care aides and assistant nurses. The descriptive of participants was calculated by means and standard deviations. Between-group comparisons were analysed with ANOVA (Table 1). The descriptive of prognostic factors and work ability by means and standard deviations (Table 1), and the relationships between variables was determined with the Spearman's rank correlation coefficient (Table 2 and 3). Prior to the regression analyses, each scale was tested and found to be reliable and valid for use in the context of home care services. The vast majority of the scales showed Cronbach's alpha levels above 0.7, indicating overall satisfactory reliability. Two scales scored slightly under the preferred value but still higher than 0.6 that is seen as the lowest acceptable score [42]. Hierarchical multiple regression analyses were used to test a priori defined prognostic model for self-efficacy (Model 1) and for work ability (Model 2) for care aides and assistant nurses separately (Tables 4 and 5). The principle behind the selection of the variables to be used in the regressions was to combine variables reflecting individual factors, and the demands and resources of the job. In the first step of the analysis, *basic background factors *(age, sex and seniority) were introduced to serve as controls. In the second step, three variables representing *job-related factors *were added: safety climate, psychosocial job demands and physical job demands. In the third step, three factors representing *individual resources *were added: degree of personal safety, personal safety behaviour and self-efficacy. The fourth and final step, involved the introduction of the single variable '*musculoskeletal wellbeing*'. A significance level of *p *< 0.10 was taken to denote statistical significance in the regressions. We used this significance level because the sample size was relatively low, making it more difficult to reach statistical significance. Using a slightly higher significance level (as advocated for exploratory studies), we are able to detect more possible practical relationships that if found theoretically sound can be tested in future studies. The largest estimate of the variance inflation factor (VIF) found was 2.6 for safety climate, while the majority of the independent variables showed levels below 1.5, indicating that multicollinearity was not a problem. The models were checked for normal assumptions for linear regression, which was found to be satisfactory [42]. All statistical analyses were performed using SPSS version 18.0.

**Table 1:** Descriptive of the included prognostic variables and the work ability for the professions

	Care aide (n = 58)	Assistant nurse (n = 79)	
	Mean, SD	Mean, SD	p^1^
1. Age, years	44.0 ± 12.6	46.5 ± 9.3	0.177
2. Sex (men/women, %)	9%/91%	6%/94%	0.614
3. Seniority, years	13.1 ± 9.4	11.6 ± 8.1	0.310
4. Safety climate, overall	3.2 ± 0.5	3.3 ± 0.4	0.474
5. Psychosocial job demands	2.5 ± 0.4	2.5 ± 0.4	0.829
6. Physical job demands	13.3 ± 2.6	13.2 ± 2.3	0.706
7. Personal safety behaviour	5.4 ± 0.9	5.5 ± 0.9	0.271
8. Degree of personal safety	3.4 ± 0.7	3.4 ± 0.6	0.891
9. Self-efficacy	4.4 ± 0.5	4.6 ± 0.4	**0.004**
10. Musculoskeletal wellbeing	4.4 ± 0.7	4.1 ± 1.0	0.096
11. Work ability	15.1 ± 2.1	15.4 ± 1.8	0.331

Dichotomous variable: Sex: Men = 1, women = 2.

^1 ^Differences between the two professions, care aides and nursing assistants, analysed with ANOVA. Significance level 0.05.

**Table 2:** Correlations between the variables, for the care aides (n = 58)

	Alpha	Scale	1	2	3	4	5	6	7	8	9	10
1. Age	.	.										
2. Sex (men/women, %)	.	.	0.22									
3. Seniority, years	.	.	**0.48**	0.23								
4. Safety climate, overall	0.96	1-4	0.19	0.07	0.13							
5. Psychosocial job demands	0.75	1-4	0.08	0.12	0.13	**-0.43**						
6. Physical job demands	.	6-20	-0.05	0.01	-0.11	-***0.29***	**0.60**					
7. Personal safety behaviour	0.85	1-7	0.14	0.10	-0.08	**0.51**	-0.23	**-0.35**				
8. Degree of personal safety	0.76	1-5	-0.11	-0.18	-0.02	**0.61**	**-0.41**	***-0.32***	***0.33***			
9. Self-efficacy	0.70	1-5	0.17	-0.09	0.15	**0.44**	**-0.34**	**-0.42**	***0.26***	**0.40**		
10. Musculoskeletal wellbeing	0.74	1-5	0.16	-0.25	0.12	***0.28***	-0.22	**-0.38**	0.25	**0.46**	**0.40**	
11. Work ability	0.73	3-17	***-0.32***	-0.13	0.11	***0.27***	***-0.33***	***-0.31***	0.17	**0.36**	**0.38**	**0.28**

Pearson's correlations (2-tailed) are significant at the **0.01 level **and at the ***0.05 level***.

Dichotomous variable: Sex: Men = 1, women = 2.

**Table 3:** Correlations between the variables, for the assistant nurses (n = 79)

	Alpha	Scale	1	2	3	4	5	6	7	8	9	10
1. Age, years	.	.										
2. Sex (men/women, %)	.	.	***0.23***									
3. Seniority, years	.	.	***0.25***	***0.26***								
4. Safety climate, overall	0.96	1-4	0.10	-0.09	-0.09							
5. Psychosocial job demands	0.66	1-4	-0.05	-0.11	0.08	**-0.46**						
6. Physical job demands	.	6-20	0.10	0.11	0.17	-0.04	**0.30**					
7. Personal safety behaviour	0.87	1-7	0.22	0.03	-0.11	**0.36**	-0.20	-0.12				
8. Degree of personal safety	0.69	1-5	0.04	0.01	-0.06	**0.41**	**-0.39**	***-0.28***	**0.30**			
9. Self-efficacy	0.63	1-5	**0.30**	-0.18	-0.06	**0.35**	**-0.32**	**-0.34**	**0.33**	**0.37**		
10. Musculoskeletal wellbeing	0.86	1-5	-0.03	-0.18	-0.15	0.08	-0.16	***-0.24***	0.03	**0.33**	0.21	
11. Work ability	0.67	3-17	0.06	0.00	-0.16	***0.24***	-0.20	***-*0.30**	***0.22***	**0.49**	**0.45**	**0.46**

Pearson's correlations (2-tailed) are significant at the **0.01 level **and at the ***0.05 level***.

Dichotomous variable: Sex: Men = 1, women = 2.

**Table 4:** The results of the prognostic model tested for the self-efficacy (model 1) and work ability (model 2), for the care aides (n = 58)

	Self-efficacy			Work ability			
	*Step 1*	*Step 2*	*Step 3*	*Step 1*	*Step 2*	*Step 3*	*Step 4*
Age	0.15	0.10	0.13	-0.46***	-0.50***	-0.54***	-0.57***
Sex	-0.15	-0.14	-0.11	-0.11	-0.10	-0.06	-0.02
Seniority, years in post	0.11	0.06	0.05	0.36**	0.34**	0.36**	0.35**
Safety climate, overall	.	0.33**	0.24	.	0.23*	0.04	0.06
Psychosocial job demands	.	-0.03	-0.01	.	-0.13	-0.15	-0.16
Physical job demands	.	-0.29*	-0.28*	.	-0.16	-0.02	0.01
Degree of personal safety	.	.	0.16	.	.	0.05	-0.01
Personal safety behaviour	.	.	-0.02	.	.	0.12	0.11
Self-efficacy					.	0.28**	0.26*
Musculoskeletal wellbeing				.	.	.	0.15
F-ratio	1.06	3.92	3.01	4.49	4.85	4.03	3.75
R^2^	0.06	0.32	0.33	0.20	0.36	0.43	0.44
R^2^adj	0.00	0.24	0.22	0.16	0.29	0.32	0.32
Significance	0.374	0.003	0.008	0.007	0.001	0.001	0.001
R^2 ^Change	.	0.26	0.01	.	0.16	0.07	0.01
F (R^2 ^Change)	.	6.45	0.52	.	4.36	1.89	1.11
Sign (R^2 ^Change)	.	0.001	0.595	.	0.008	0.143	0.298

The independent variables entered to the model in steps are basic background factors (step 1), job-related factors (step 2), individual resources (step 3), and musculoskeletal wellbeing (step 4)

Significance levels: *p < 0.10, **p < 0.05, ***p < 0.01 Regression coefficients shown are beta coefficients.

Dichotomous variable: Sex: Men = 1, women = 2.

**Table 5:** The results of the prognostic model tested for the self-efficacy (model 1) and work ability (model 2), for the assistant nurses (n = 79)

	Self-efficacy			Work ability			
	*Step 1*	*Step 2*	*Step 3*	*Step 1*	*Step 2*	*Step 3*	*Step 4*
Age	0.38***	0.36***	0.33***	0.10	0.09	-0.04	-0.05
Sex	-0.24**	-0.22**	-0.23**	0.03	0.07	0.13	0.18*
Seniority, years in post	-0.09	-0.01	-0.01	-0.19	-0.13	-0.13	-0.10
Safety climate, overall	.	0.22*	-0.11	.	0.22*	0.04	0.06
Psychosocial job demands	.	-0.13	-0.11	.	0.01	0.11	0.13
Physical job demands	.	-0.30***	-0.24**	.	-0.28**	-0.11	-0.08
Degree of personal safety	.	.	0.16	.	.	0.36***	0.26**
Personal safety behaviour	.	.	0.12	.	.	-0.01	0.02
Self-efficacy				.	.	0.34***	0.32***
Musculoskeletal wellbeing				.	.	.	0.32***
F-ratio	4.86	6.86	5.84	0.95	2.34	4.32	5.42
R^2^	0.16	0.36	0.40	0.04	0.16	0.36	0.44
R^2^adj	0.13	0.31	0.33	0.00	0.09	0.28	0.36
Significance	0.004	0.000	0.000	0.421	0.040	0.000	0.000
R^2 ^Change	.	0.20	0.04	.	0.13	0.20	0.08
F (R^2 ^Change)	.	7.58	2.13	.	3.63	7.01	10.1
Sign (R^2 ^Change)	.	0.000	0.127	.	0.017	0.000	0.002

The independent variables entered to the model in steps are basic background factors (step 1), job-related factors (step 2), individual resources (step 3), and musculoskeletal wellbeing (step 4).

Significance levels: *p < 0.10, **p < 0.05, ***p < 0.01 Regression coefficients shown are beta coefficients.

Dichotomous variable: Sex: Men = 1, women = 2.

## Results

### Response rate and personal descriptive of the participants

The 137 (46%) participants had a mean age of 45 years, 93% were women and 42% (n = 58) were care aides while the others (n = 79) were assistant nurses. Personal descriptive and descriptive of the included prognostic variables and the work ability, for the two professions are presented in Table 1. The assistant nurses reported significantly higher self-efficacy (*p = *0.004) than the care aides, but there was no significant difference between the professions in regard to any of the other variables studied (Table 1). As discussed in the introduction, separate regression analyses were performed for the two groups to establish whether they were similar or different for each of the prognostic variables studied.

### Regression models

The regression analyses assessing the several independent variables associations with self-efficacy (Model 1) and work ability (Model 2) were performed separately for the care aides and assistant nurses. The results are presented in Tables 4 and 5.

### Model 1: Prognostic factors for self-efficacy

For the care aides, the regression analysis performed with self-efficacy as the dependent variable was not significant until the *second step*, indicating that the background factors (age, sex and seniority), used as controls, were not associated with self-efficacy. When the variables representing job-related factors were entered, it was revealed that the overall safety climate and physical job demands significantly contributed to the explained variance: the added variables explained 26% (*p = *0.001), and the overall model had an R^2^adj of 0.24 (*p = *0.003). The third step did not attain significance, indicating that neither perceived degree of personal safety nor personal safety behaviour were linked to self-efficacy for this group. Thus, for care aides, a higher self-efficacy was exhibited by those who reported a strong safety climate and by those who perceived less physical exertion in their job (Table 3).

The regression analysis for the assistant nurses attained significance during the *first step*, where age and sex significantly affected self-efficacy: the background factors explained 16% (*p = *0.004) of the variance. When the job-related factors were entered in the *second step*, the safety climate and the physical job demands provided a significant contribution to self-efficacy, and the variables added explained an additional 20% (*p *< 0.001). In the third step, the change was not significant. Thus, the overall model had an R^2^adj of 0.31 (*p *< 0.001), implying that among assistant nurses, a higher self-efficacy was associated with being older, being a man, perceiving less physical exertion in their job and being in a stronger safety climate (Table 4).

### Model 2: Prognostic factors for work ability

For the care aides, the analysis performed with their work ability as the dependent variable was already significant in the *first step*, where age and seniority were found to be significantly associated with work ability, and the background factors that were added explained 20% (*p = *0.007) of the variance. When the variables representing specific job-related factors were entered in the *second step*, the safety climate contributed significantly to the work ability and the variables that were added explained a further 16% (*p *= 0.008). The overall model had an R^2^adj of 0.29 (*p *= 0.001). In the third and fourth steps, the changes were not significant (*p = *0.143), indicating that individual resources and musculoskeletal wellbeing were not linked with work ability in this group. Hence, among the care aides, a higher work ability was related with being of a younger age, having longer seniority and being in a stronger safety climate (Table 3).

For assistant nurses, the model for explaining variations in work ability did not achieve significance until the *second step*, indicating that the background factors were not linked to work ability. In the *second step*, overall safety climate and physical job demands provided significant contributions to work ability, and the job-related factors that were added explained an additional 13% (*p *= 0.017) of the variance. In the *third step*, the degree of personal safety and self-efficacy provided significant contributions and explained a further 20% (*p <*0.001), while the job-related factors lost significance. In the *fourth step*, musculoskeletal wellbeing contributed significantly and explained an additional 8% (*p = *0.002) of the variance; sex also appeared to be a significant contributor. The R^2^adj for the overall model was 0.36, (*p *< 0.001). This implies that the work ability was positively associated with being a woman and by reporting a higher degree of personal safety, higher self-efficacy in relation to work and safety, and higher musculoskeletal wellbeing (Table 4).

## Discussion

### Differences and similarities between home care aides and assistant nurses

The prognostic factors for work ability as well as for self-efficacy for care aides and assistant nurses were assessed separately. Of the variables used in the models, only the level of perceived self-efficacy differed significantly between the two professions, being higher for the assistant nurses. In both professions, a high frequency of musculoskeletal symptoms was reported, with only about a quarter of the participants having no musculoskeletal complaints. However, the overall results of the hierarchical multiple regression analyses indicated that the independent variables used in the models made different contributions for the care aides and assistant nurses.

### Prognostic factors for work ability

In this study, 'work ability' reflected the staff's own prediction about their ability to perform their present work in a further 2 years because of their state of health, and their work ability in relation to the specific demands posed by their job. Among care aides, higher work ability was associated with being younger, having a relatively long experience of working within the home care services, and reporting a 'stronger' safety climate. As professional skill and experience develop over time, it can be seen how a greater seniority would provide a greater ability to perform the tasks required more efficiently [43]. Accumulated seniority could also reflect the underlying health of the workers in question, as it is possible that some former workers could have changed jobs to work in another field for health reasons. Earlier findings revealed positive feedback to be important for work ability [33,44]. A 'safety climate' specifies that the quality of the feedback and support received from managers and peers, should, e.g., be directed towards the need to prioritise healthy and safe behaviour at work, irrespective of the desire to provide high quality service.

In the model for explaining the work ability of assistant nurses, a greater ability was exhibited by being a woman and by reports of high self-efficacy, degree of personal safety and musculoskeletal wellbeing. This can be viewed as a proactive attitude driven by the desire of workers to remain free of injury, a belief in having the skill to, and taking, preventive actions to avert potential threats [45]. The fact that women with physically demanding jobs run a higher risk of developing WMSDs has been shown in previous studies [46,47]. The education required in training to be an assistant nurse may instil a greater awareness of one's own health and needs to take preventive actions, reflecting functional optimism [7,8]. Holding an active belief in one's own ability to shape one's work context and having high quality communication are considered to influence participation in proactive safety activities [30], and to provide a buffer against WMSDs [46]. Unexpectedly, the degree of compliance with personal protection regulations did not contribute significantly in any of the regression models. It is possible, that in contexts where the environment and the tasks required vary considerably, and in which all clients are unique, it is not possible to standardise all of the processes [48]. Instead, staff self-efficacy at the identification and implementation of safe work practices in varied and even unexpected situations may be more influential than an ability to work within regulations [10]. Accordingly, there could be reasons to promote workers' knowledge, self-efficacy and ability to identify alternative courses of action when encountering environmental demands. Hence, it is important to consider the actual opportunities that are allowed to the individual or to groups of workers within an organisation to exert control [14,17,18]. As the 'safety climate' includes the management's ability to increase workers' empowerment, this is an interesting resource to explore further within the context of home care services.

### Prognostic factors for self-efficacy

Perceived self-efficacy in relation to work and safety was focused, such as handling most situations at work, adjusting work tasks and to influence safety. For both professions, the models for explaining self-efficacy in relation to work and safety showed that higher self-efficacy was evidenced among those who perceived a low level of physical exertion and who reported a stronger safety climate. Furthermore, for assistant nurses, being older and being a man were also linked with higher self-efficacy beliefs. This is in line with recent findings in medical care, which showed that, within the same workplace, those with a low status and with a low degree of authority to influence the job content have the highest overall physical exposure levels [30]. Earlier research in home care services showed that group solidarity, collegial support and being acknowledged for the performance of one's home care job are important for the job satisfaction [44]. Therefore, the prognostic value of the safety climate is important. Safety climate indicates whether the social influences of the manager and colleagues are likely to encouraging workers to perform their job safely [19,21,22]. According to Bandura [43], self-efficacy can be developed from the experience of having had successful experiences of an action previously and through the influence of the social environment, for example, through other people acting as role models for successful practice, and as a result of verbal persuasion. Thus, the actual job content, situational constraints and role expectations at work may influence the development of self-efficacy. For example, if the person performing the work encounters respect in his or her cooperation and in his or her communication with others (e.g., with clients, medical care staff and management). It is plausible that the education provided during training to become an assistant nurse helps to develop skills that are reflected in a higher self-efficacy where work and safety are concerned. Research on female-dominated workplaces confirmed that climate, power structures and group compositions determine the power distribution among members [29]. Furthermore, men and individuals with higher professional and life experiences are often allocated a higher status [31]. Inequality between the sexes concerning their authority and career opportunities have been revealed within the home care services [34]. Why being older and being a man were prognostic for stronger self-efficacy in nursing assistants, but not in care aides, are interesting. There is a need of further analysing each profession's opportunities for control and self-efficacy in relation to influence over their work content and conditions.

### Practical implications

We have described a theoretical model, adopted from health psychology. The strength of this model is that it provides an understanding of the chain of interacting factors leading to health and work ability. We modified the model to some extent, by specifying work place contextual factors according to the literature, and used these factors in our regression models. In the first step of the regressions, we added personal background factors that are not changeable. In the second step, the perceptions of job-related factors were added; these may be possible to modify by workplace interventions. Then, variables relating to individual resources (within the person) were added, degree of personal safety, self-efficacy and personal safety behaviour. These can be changed. Musculoskeletal wellbeing was selected as an important health variable. From a motivational and behavioural change perspective, all these factors are important factors to consider for both care aides and assistant nurses. In further research we want to develop the model (Figure 1) by including factors not yet studied. In this article we have only addressed work place relevant variables, and not specifically included leisure time, cultural or gender factors, which could be seen as blind spots which need to be further studied. Also attitudes, subjective norms and specific motivational factors need to be further acknowledged. Notably was that in both professions psychosocial job demands and personal safety behaviour did not contribute to the explained variance in work ability. Safety behaviour has not been studied earlier in relation to work ability. However, psychosocial job demands often contribute to work ability according to earlier research.

The work ability promoting factors contributed differently to work ability in the two professions, as shown by the results by the regression analyses (Tables 4 and 5). This is new knowledge and motivates a focus on these specific factors in each profession. When examining the B values (unstandardized regression coefficients), it is clear that the significant results also imply practical implications. Self-efficacy emerged as an important prognostic factor for better work ability among assistant nurses, with a B value of 1.6. Also musculoskeletal wellbeing (B value of 0.6) and personal safety perceptions (B values of 0.07) contributed, but not as much as expected. In the care aides, the non-changeable factors age and seniority were prognostic for better work ability. In this group also the safety climate emerged as important for work ability, with a B value of 1.0. The shared prognostic factors for self-efficacy for the two professions were safety climate and physical job demands, in line with theory. For the nursing assistants, self-efficacy was also associated with being of older age and a man. All these factors are important to acknowledge in practice and in further research. This implies that, for both professions, work place interventions to promote a sustainable work ability need to focus on potentially modifiable factors, such as self-efficacy, the safety climate, physical job demands and musculoskeletal wellbeing.

Effective interventions could for example be to strengthen the home care units' safety climate by introducing organisational and social support for safe work practices, focusing on the leadership and on opportunities for work place meetings to discuss shared job values and priorities [49]. This can in turn augment the individual's self-efficacy beliefs, in line with the reasoning of reciprocal interaction [8,11-13]. A stronger safety climate also implies a safer environment. Further, perceived physical job demands can be reduced by on the job training in ergonomics, including good client transfer techniques and by physical capacity training [50,51]. By viewing 'work ability' as reflective of the balance between a person's resources and the demands of their work, it is obvious that interventions need to act on different levels and be performed in parallel.

### Strength and Weakness of our study

The data for this study were derived from a cross-sectional survey, restraining us from making any conclusions about the causal associations. There are always concerns associated with which independent variables are capable of reflecting the important associations, and therefore which ones to choose. The response rate of 46% could imply a possible selection bias, which is why the results should be interpreted with some caution. However, non-responders were significantly younger (mean age 41) but did not differ from the participants in terms of their age range, sex or profession. In addition, the degree of self-reported work ability and job demands were in line with earlier research on a similar population [52]. It has been emphasised, that in research where people are engaged in social relations, the results also need to be analysed from a contextual perspective to visualise any imbalance in power [53]. It is possible that a different number, or other participants, would have agreed to take part in this research if the recruitment had by-passed their supervisor. Those who perceived a higher commitment to good health and safety practices might also have been more inclined to participate in this research. The findings represent home care workers having worked in the same unit for the last 6 months. This criterion was an attempt to ensure that the measure of the safety climate were representative of the shared perceptions of the members of a home care unit. The levels reported in some variables could, to some extent, has been influenced by recall biases, social expectations or protests. There might also be a healthy worker selection effect for instance having a relatively long experience of working within the home care services.

Thus, this research can be considered as an explorative study that has identified possible modifiable factors associated with a better work ability. We used 10 independent variables in the models, which is close to the highest acceptable number (given that we should have at least 5 observations per variable) [42]. A majority of the associations can be explained theoretically, which is supporting the findings. Independent variables making different contributions for work ability and self-efficacy in the two professions ought to be considered in future practice and research. To verify the results, the model needs to be examined in greater depth in a larger scale investigation.

## Conclusions

The intermediate factors contributed differently to work ability in the two professions. Self-efficacy, personal safety and musculoskeletal wellbeing were important for the assistant nurses, while the work ability of the care aides was associated with the safety climate, but also with the non-changeable factors age and seniority. All these factors are important to acknowledge in practice and in further research. Proactive workplace interventions need to focus on potentially modifiable factors such as self-efficacy, safety climate, physical job demands and musculoskeletal wellbeing.

## Competing interests

The authors declare that they have no competing interests.

## Authors' contributions

AL, LK and GG participated in the development of the study design. AL was responsible for the data collection and for drafting the manuscript. AL and MW performed the statistical analysis, and MW, LK and GG read and corrected draft versions of the manuscript. All authors read and approved the final manuscript.

## Pre-publication history

The pre-publication history for this paper can be accessed here:

http://www.biomedcentral.com/1471-2474/13/1/prepub
